# Identification of diagnostic genes for myocardial ischemia reperfusion injury associated with metabolic syndrome through the integration of bioinformatics analysis, molecular docking and experimental validation

**DOI:** 10.3389/fimmu.2025.1567572

**Published:** 2025-05-29

**Authors:** Shufang Liu, Yan Zhang, Yanan Zhao, Ping Wu, Shouyuan Tian, Han-Qing Pang, Zhifang Wu, Sijin Li

**Affiliations:** ^1^ Department of Nuclear Medicine, First Hospital of Shanxi Medical University, Taiyuan, China; ^2^ Collaborative Innovation Center for Molecular Imaging of Precision Medicine, Shanxi Medical University, Taiyuan, China; ^3^ School of Anesthesiology, Shanxi Medical University, Taiyuan, China; ^4^ Department of Anesthesiology, Shanxi Provincial People’s Hospital, Taiyuan, China; ^5^ Institute of Translational Medicine, School of Medicine, Yangzhou University, Yangzhou, China; ^6^ The Key Laboratory of the Jiangsu Higher Education Institutions for Integrated Traditional Chinese and Western Medicine in Senile Diseases Control, Yangzhou University, Yangzhou, China

**Keywords:** myocardial ischemia reperfusion injury, metabolic syndrome, machine learning, molecular docking, immune infiltration

## Abstract

**Background:**

Metabolic dysregulation in metabolic syndrome (MetS) exacerbates myocardial ischemia-reperfusion injury (MIRI). This study aimed to identify diagnostic biomarkers and therapeutic candidates for MetS-associated MIRI.

**Methods:**

Three MIRI and two MetS datasets from GEO were analyzed using differential expression analysis, WGCNA, and machine learning (LASSO/SVM-RFE). Hub genes were validated via qRT-PCR in hypoxia-induced H9C2 cells. Drug candidates were predicted via PPI networks, CTD, and molecular docking, followed by experimental evaluation of dexamethasone.

**Results:**

Five hub genes—DAK, GTF3C5, KCNMB1, TRAF1, and ZNF692—were identified, with distinct expression patterns (DAK/GTF3C5 downregulated; KCNMB1/TRAF1/ZNF692 upregulated). These genes were enriched in immune-related pathways, and their diagnostic performance was robust (AUCs: 0.875–0.969). Dexamethasone downregulated KCNMB1/TRAF1/ZNF692 and reduced apoptosis in H9C2 cells.

**Conclusion:**

This study reveals immune-metabolic dysregulation as a key driver of MetS-MIRI, proposes five biomarkers for diagnosis, and highlights dexamethasone as a promising therapeutic candidate.

## 1 Introduction

Myocardial ischemia-reperfusion injury (MIRI) is one of the common pathogenic mechanisms in patients with acute coronary syndrome (ACS) and myocardial infarction (MI), and remains a major challenge in the treatment of cardiovascular diseases (1–3). Although emergency procedures such as percutaneous coronary intervention (PCI) have significantly reduced the incidence of cardiovascular events by successfully restoring blood flow, the heart faces multiple injuries after reperfusion, including acute oxidative stress, inflammatory responses, and cell death (4, 5). Notably, these injuries are exacerbated in patients with metabolic syndrome (MetS), where genetic predispositions impair endogenous antioxidant defenses, leading to amplified reactive oxygen species production during reperfusion (6). This process not only exacerbates functional damage to the heart but may also trigger long-term cardiac remodeling, ultimately leading to heart failure and other life-threatening conditions (7). Therefore, how to effectively prevent and treat MIRI is a critical issue that urgently needs to be addressed in the field of clinical cardiovascular diseases.

MetS is a group of pathological conditions characterized by multiple metabolic abnormalities, including obesity, insulin resistance, diabetes, hypertension, and hyperlipidemia (8, 9). MetS is considered a risk factor for various chronic diseases, including cardiovascular diseases, type 2 diabetes, stroke, and certain types of cancer (10). Emerging studies reveal that MetS-associated genetic variants disrupt adipokine signaling, thereby promoting systemic insulin resistance and pro-inflammatory cytokine release (e.g., TNF-α, IL-6), which synergistically aggravate myocardial inflammation during reperfusion (11). In recent years, an increasing number of studies have shown a significant correlation between MetS and MIRI (12, 13). This phenomenon is linked to the exaggerated inflammatory response and oxidative stress in MetS patients. Furthermore, MetS can cause abnormal fatty acid metabolism, increasing the oxidative burden on the myocardium, thereby exacerbating reperfusion injury (14, 15). For instance, defective CPT1B alleles in MetS impair mitochondrial fatty acid β-oxidation, resulting in lethal arrhythmias post-reperfusion (16).

Although the mechanisms underlying MIRI have been extensively studied, existing treatment options still face significant challenges. Traditional therapies mainly include antioxidant treatment, anti-inflammatory treatment, and cyto-protection, but these methods have shown limited efficacy in clinical applications (17, 18). Some drugs can alleviate oxidative stress and inflammatory responses after myocardial reperfusion, but due to their significant side effects or lack of long-lasting effects, they have not become standard treatment options for MIRI (19). Importantly, these limitations may stem from a failure to address the genetic-epigenetic crosstalk in MetS. For example, conventional antioxidants cannot rescue NRF2 promoter methylation-induced suppression of phase II detoxifying enzymes in MetS patients, leaving cardiomyocytes vulnerable to ROS bursts (20). Additionally, pathophysiological processes such as cell apoptosis, mitochondrial dysfunction, and endothelial damage after myocardial reperfusion remain therapeutic challenges (21, 22). Studies indicate that after myocardial ischemia-reperfusion, myocardial cells undergo varying degrees of apoptosis, necrosis, and autophagy through multiple pathways, processes that are difficult to effectively inhibit with conventional drug interventions (23–25). Therefore, the development of new therapeutic strategies, particularly targeted drugs aimed at metabolic dysregulation and cellular damage, has become a current research focus.

The occurrence of MetS is closely associated with mutations or abnormal expression of multiple genes, and the role of these genetic alterations in MIRI has become a focus of research (26). Studies suggest that changes in genes related to lipid and glucose metabolism, such as insulin receptor substrates (IRS) and fatty acid synthase (FAS), in MetS may affect the metabolic state of myocardial cells, thereby influencing the extent of MIRI (27–29). Research has shown that mutations in the IRS-1 gene lead to the development of MetS, while simultaneously increasing the severity of MIRI, an effect closely related to disruptions in fatty acid metabolism and intracellular energy supply (30, 31). Additionally, the widespread inflammatory response in MetS patients is also linked to the abnormal expression of certain key genes (32). Studies indicate that levels of inflammatory cytokines such as TNF-α and IL-6 are elevated in the serum of MetS patients, and the persistent activation of these cytokines exacerbates myocardial injury during myocardial ischemia-reperfusion by promoting apoptosis and local inflammation (33, 34). With the advancement of genomics and transcriptomics technologies, more key genes related to MetS have been discovered, providing new perspectives for further exploring the relationship between MetS and MIRI (35).

In recent years, significant progress has been made in the development of small molecule drugs for the treatment of cardiovascular diseases. Compared to traditional macromolecular drugs, small molecule drugs offer better cell permeability, lower toxicity, and stronger targeting capabilities, making them highly promising for the treatment of MIRI. Our research specifically focuses on designing dual-target inhibitors that simultaneously rectify MetS-related genetic defects (e.g., *PPAR-γ* agonists to restore fatty acid oxidation) and mitigate MIRI-specific damage (e.g., RIPK1 inhibitors to block necroptosis), thereby breaking the vicious cycle between metabolic dysfunction and reperfusion injury. As the mechanisms underlying MetS and MIRI are increasingly understood, more research has focused on developing small molecules targeting these mechanisms, particularly those capable of regulating metabolic pathways, reducing inflammation, mitigating oxidative stress, and repairing damaged cells. Such small molecule drugs are expected to become effective therapeutic options for MIRI in the future.

## 2 Methodology

### 2.1 Collection and processing of microarray data

The raw expression profile datasets for MIRI, including GSE6381, GSE108940, and GSE160516, were obtained from the GEO database (https://www.ncbi.nlm.nih.gov/geo/). The raw expression profile datasets for peripheral blood mononuclear cells (PBMCs) from MetS patients, GSE98895 and GSE200744, were also sourced from the GEO database. Detailed descriptions of the datasets are provided in **Table 1**.

**Table 1:** Basic information of GEO datasets used in the study.

GSE series	Tissue	Organism	Sample size		Platform
			Control	MIRI	
GSE6381	Heart	Homo sapiens	4	8	GPL96
GSE108940	Heart	Mouse	6	6	GPL7202
GSE160516	Heart	Mouse	4	12	GPL23038

			Control	Mets	
GSE98895	PBMC	Homo sapiens	20	20	GPL6947
GSE200744	PBMC	Homo sapiens	33	25	GPL28038

### 2.2 Differential expression analysis

Differential expression analysis of genes (DEGs) was performed using the **limma** package. DEGs between the normal group and myocardial ischemia-reperfusion group in the GSE6381 dataset, as well as DEGs between the normal group and MetS patients in the GSE98895 dataset, were selected using a threshold of P.Val < 0.05. The results of DEGs were visualized through volcano plots and heatmaps, which were generated using the ggplot2 and pheatmap packages.

### 2.3 Weighted gene co-expression network analysis

The WGCNA package in R was used to construct a gene co-expression network and identify modules related to MIRI in the GSE6381 dataset and modules related to MetS in the GSE200744 dataset. The soft threshold power (β) was selected using the **pickSoftThreshold** function, with a fit index >0.9 as the criterion. The adjacency matrix was calculated using the adjacency function, and then converted into a Topological Overlap Matrix (TOM) using the TOMsimilarity function. The mergeCloseModules function was used to merge highly correlated modules of feature genes. The module feature relationships were displayed and visualized using heatmaps. Additionally, the module membership (MM) and gene significance (GS) scores of the modules were evaluated to interpret module significance.

### 2.4 Enrichment analysis

We further conducted functional enrichment analysis of the selected genes using the Microbioinformatics platform (https://www.bioinformatics.com.cn/). First, Gene Ontology (GO) analysis was performed to assess enrichment in three categories: Biological Process (BP), Molecular Function (MF), and Cellular Component (CC), to reveal the biological functions of these genes and their potential mechanisms of action within the cell. Additionally, Kyoto Encyclopedia of Genes and Genomes (KEGG) pathway enrichment analysis was carried out. KEGG analysis helps identify the association between genes and specific biological pathways.

### 2.5 Machine learning

Gene expression data was filtered using the Least Absolute Shrinkage and Selection Operator (LASSO) method. LASSO reduces redundant features by introducing an L1 regularization term, selecting the most important genes for model prediction. This method effectively performs variable selection and enhances the interpretability of the model, ensuring that the selected genes are closely related to the research objectives. Subsequently, the Support Vector Machine-Recursive Feature Elimination (SVM-RFE) method was used to further refine the selected genes. SVM-RFE recursively eliminates irrelevant features and evaluates the classification ability of genes using a SVM, progressively optimizing the feature set to identify the hub genes with the greatest classification ability.

### 2.6 The construction of nomogram

The nomogram function from the rms package was used to generate a nomogram. In the nomogram, each variable corresponds to a score axis, and the total score axis is used to predict the final risk value. The expression comparison of the three characteristic genes in the training cohort (GSE6381) and validation cohort (GSE108940 and GSE160516) were investigated. The receiver operating characteristic (ROC) curve was constructed using the “pROC” package to evaluate the diagnostic performance of the signature genes and nomogram.

### 2.7 Immune cell infiltration analysis

The CIBERSORT algorithm was used for the quantitative analysis of immune cell infiltration in the samples through the CIBERSORT R script. CIBERSORT utilizes a gene expression signature matrix of 22 known immune cell subtypes and applies linear support vector regression (SVR) to deconvolve the expression matrix of human immune cell subtypes, thereby assessing the relative proportions of the 22 immune cell types in each sample. By comparing the differences in immune cell infiltration between the MIRI group, MetS group, and control group, we further evaluated the changes in immune cells. The correlation between immune cells was visualized using the corrplot package.

### 2.8 Protein-protein interaction network analysis and cluster analysis

The key genes were analyzed through the STRING database (https://www.stringdb.org) with a medium confidence score > 0.4. The protein-protein interaction (PPI) network was imported into Cytoscape software (version 3.9.0) for visualization, and the cytoHubba module was used to predict the gene clusters with the highest scores.

### 2.9 Comparative toxicogenomics database drug prediction analysis

Based on the gene-drug interaction network in the CTD database, and by combining the mechanisms of action of the drugs with gene expression patterns, the response of each drug to the key genes was predicted. By comparing the effects of different drugs on the target genes, their potential efficacy and drug sensitivity in specific diseases were evaluated.

### 2.10 Molecular docking

The full-length sequences of KCNMB1, ZNF6921, and TRAF1 were obtained from the PDB database, and their structures were predicted using AlphaFold. Additionally, the 3D structure of Dexamethasone (CAS: 50-02-2) in SDF format was retrieved from the PubChem database. All protein and molecule files were converted to PDBQT format, and polar hydrogen atoms were added. The docking pocket was set as a cubic pocket of 40 Å × 40 Å × 40 Å, with a grid spacing of 0.05 nm. To assess the binding affinity of the candidate drug to its target, molecular docking analysis was performed. The binding poses of the candidate drug with the protein were obtained using Autodock Vina 1.2.2, along with the binding energies of the best protein-ligand complex interactions. The binding interface of the protein-ligand complex was systematically analyzed using PLIP and LigPlus, and interaction details were further supplemented using pyMOL 2.5 software.

### 2.11 Construction of MIRI cell model

The H9c2 cells were purchased from Procell and cultured under hypoxic conditions (1-3% oxygen) for 24 hours to simulate ischemia. Maintain a parallel normoxic control group, i.e. untreated cells at 21% oxygen concentration, throughout the entire experiment. After hypoxia, the cells were transferred to normal oxygen conditions (21% O_2_) to simulate the reperfusion process, with reoxygenation typically lasting 2-24 hours. All experimental readouts were normalized to time-matched normoxic controls to distinguish hypoxia/reoxygenation-specific effects. This model can simulate various physiological processes involved in myocardial ischemia and reperfusion, such as oxidative stress, apoptosis, and inflammatory responses.

### 2.12 RT-qPCR validation of hub gene expression

Total cellular RNA from MIRI was extracted using RNA extraction reagent (Trizol) according to the manufacturer’s instructions. cDNA synthesis and quantitative real-time PCR (qRT-PCR) were performed following the manufacturer’s guidelines. The expression of hub genes was represented as 2^-△△Ct^ relative to Gapdh for mRNA quantification. The rat homologous genes primer sequences are listed in **Table 2**.

**Table 2:** Rat homologous genes primer sequences.

Gene Name	Primer Sequence
Tkfc	F:5’-CCTCTATAGCTCCAGGCGTC-3’
	R:5’-CTGAGTTGCTGCAGGGTTGA-3’
Gtf3c5	F:5’-TTCACCGCAATGATGGGACA-3’
	R:5’-TGTTGGAGAACAGAGCAGGC-3’
Kcnmb1	F:5’-TAGAGCTCCAAGGCCTGACT-3’
	R:5’-ACAGTCATTTAGTTCCCTGGGT-3’
Traf1	F:5’-AGGGCTGGTCCTCTACTTTACT-3’
	R:5’-GGACAGATCCGTTCCTCATCG-3’
Zfp692	F:5’-ACCAGTACCTGCAACTCTGC-3’
	R:5’-TCACAAGTCGGATTCGGAGC-3’
Gapdh	F:5’-TCTCTGCTCCTCCCTGTTC-3’
	R:5’-ACACCGACCTTCACCATCT-3’

### 2.13 Flow cytometry analysis of MIRI cell apoptosis

Flow cytometry was used to analyze the extent of cell apoptosis in H9c2 cells after MIRI. The cells were first collected and washed with PBS, followed by staining using the Annexin V-FITC/PI apoptosis detection kit. Annexin V-FITC was used to label early apoptotic cells, while PI (propidium iodide) stained late apoptotic or necrotic cells. After staining, the samples were analyzed by flow cytometry using the BD FACSCanto II for data acquisition.

### 2.14 Statistical analysis

All statistical analyses were performed using R software 4.3.1. Wilcox or Student’s t-test was implemented to analyze the differences between the two groups. Pearson’s or Spearman’s correlation test was conducted to determine the correlation between the variables. When p <0.05, the observed difference was considered statistically significant.

## 3 Results

### 3.1 Data processing, DEGs identification, and GSEA enrichment analysis

**Figure 1:**
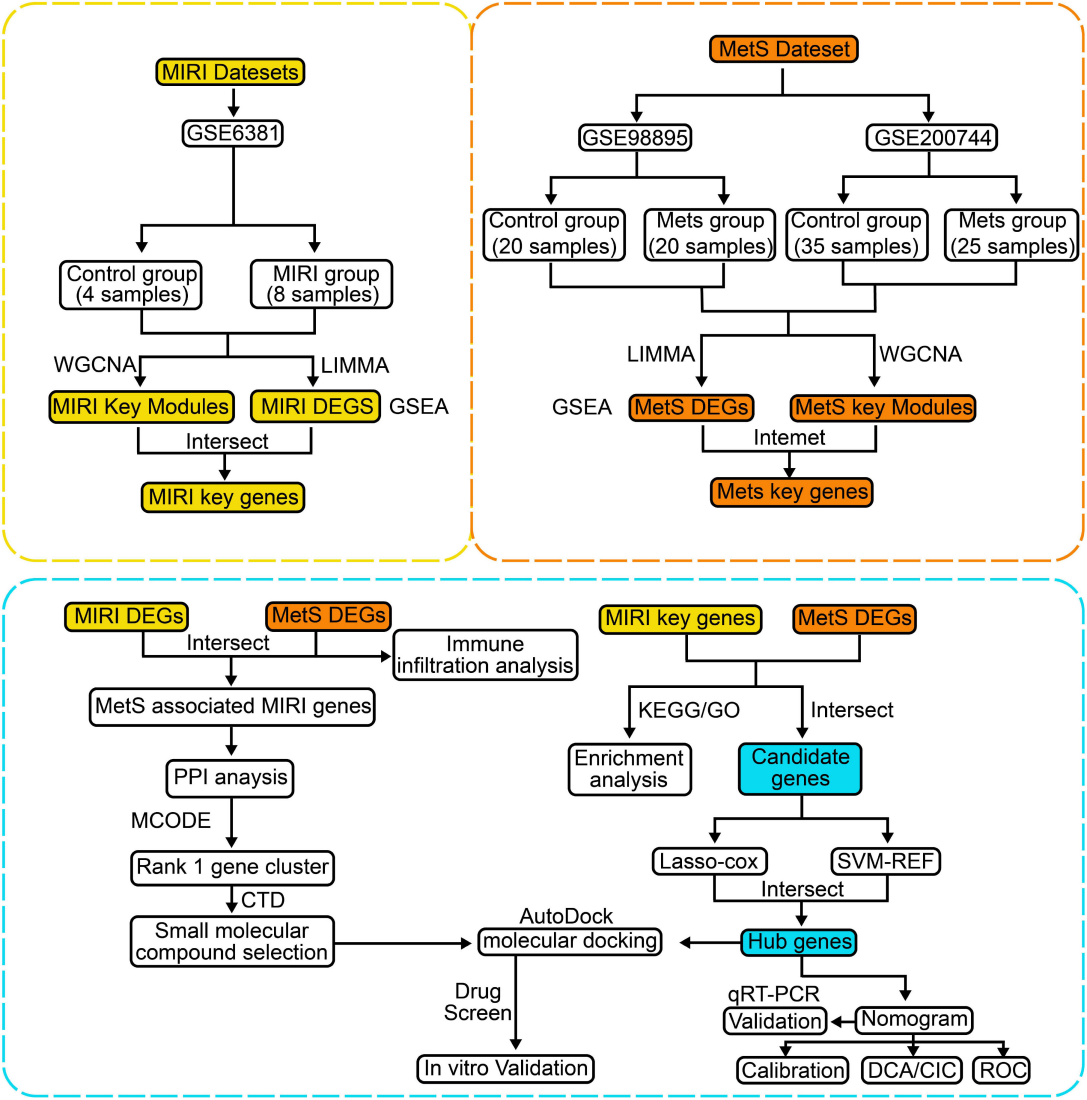
The detailed workflow of this study.

**Figure 2:**
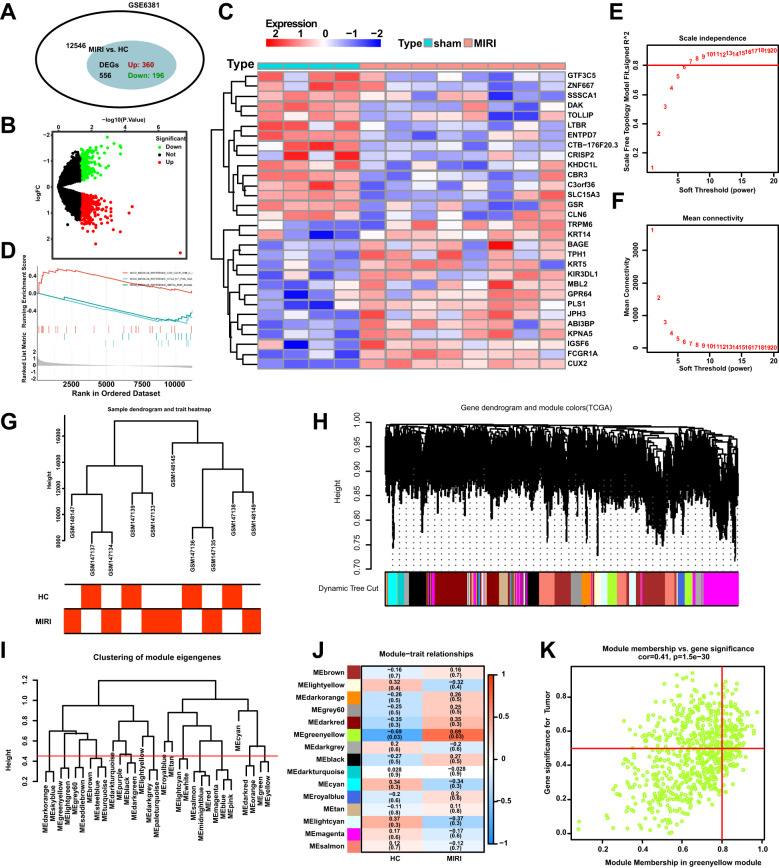
DEGs and WGCNA enrichment analysis of the MIRI genome. **(A)** Venn diagram showing the number of DEGs between the MIRI and control groups. **(B)** Volcano plot of differentially expressed genes (DEGs) in the MIRI group. **(C)** Heatmap illustrating the expression patterns of MIRI DEGs. **(D)** GSEA enrichment analysis results for MIRI DEGs. **(E)** Relationship between scale independence and soft threshold in the WGCNA network topology analysis. **(F)** Relationship between average connectivity and soft threshold in the WGCNA network topology analysis. **(G)** Hierarchical clustering tree of MIRI samples and modules. **(H)** Correlation analysis between the MIRI gene network and module content. **(I)** Hierarchical clustering of MIRI module genes. **(J)** Heatmap showing the correlation between MIRI modules and clinical features. **(K)** Scatter plot illustrating the relationship between module membership (MM) and gene significance (GS) in MIRI.

The flowchart of the bioinformatics analysis is shown in **Figure 1**. To analyze the genomic changes in MIRI, differential expression analysis was performed. In the GSE6381 dataset, there were 200 upregulated genes and 156 downregulated genes between the normal group (HC) and MIRI group (**Figure 2A**). Volcano plots and heatmaps were used to visualize the expression patterns of DEGs in MIRI (**Figures 2B, C**). GSEA analysis revealed upregulation of the KEGG MEDICUS REFERENCE CCR CXCR GNB G PI3K RAC SIGNAI pathway, while the KEGG MEDICUS REFERENCE KITLG KIT PI3K SIGNALING PATHWAY and KEGG MEDICUS REFERENCE WNT5A ROR SIGNALING PATHWAY were downregulated (**Figure 2D**). To further explore the key genes involved in MIRI, we conducted a WGCNA analysis to identify critical modules associated with MIRI. Based on scale independence and average connectivity, a soft threshold of 7 was applied (**Figures 2E, F**). The clustering dendrograms for MIRI and control groups are shown in **Figures 2G, H**. To further evaluate these modules, we selected a cutting line for the module dendrogram and merged modules with a distance less than 0.45, resulting in a total of 15 co-expression modules (**Figures 2I, J**). Spearman’s correlation coefficients were performed to plot the module-trait relationship and assess the correlation between each module and MIRI diagnosis (**Figure 2K**). The green-yellow module showed the highest positive correlation with MIRI (720 genes, r = 0.69, P = 0.03) and was selected as the most relevant module for MIRI. Specific gene modules were closely associated with MIRI and hold potential biomarker value, further advancing our understanding of the functional roles of genes in MIRI.

**Figure 3:**
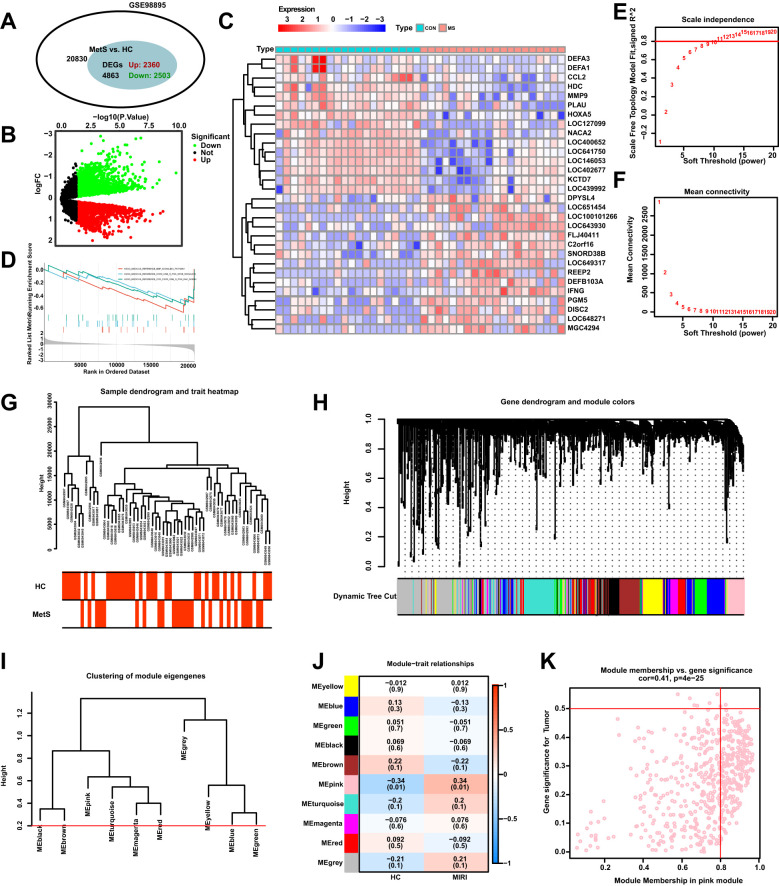
Differential expression analysis of MetS transcriptomic data and WGCNA module selection. **(A)** The Venn diagram illustrates the total number of genes and the number of DEGs associated with the MetS-related GSE98895 dataset. **(B)** The volcano plot depicts the expression patterns of DEGs in the MetS-related GSE98895 dataset. **(C)** Heatmap showing the expression patterns of significantly upregulated or downregulated DEGs in the MetS dataset, with each row corresponding to a DEG and each column corresponding to a MetS case or control sample. **(D)** GSEA enrichment analysis of DEGs in the GSE98895 dataset. **(E)** Identification of the optimal β value using a scale-free topology model, with the scale independence and **(F)** average connectivity analysis indicating β = 11 as the chosen soft threshold. **(G)** Hierarchical clustering tree of MetS and control samples. **(H)** Gene co-expression modules represented by different colors under the gene tree. **(I)** Heatmap of the adjacency of characteristic genes. **(J)** Heatmap showing the relationship between module characteristic genes and MetS. **(K)** Correlation plot between module membership and gene significance in the pink module.

We employed differential analysis and WGCNA to investigate the genomic alterations in Mets. In **Figure 3A**, the GSE98895 dataset revealed 2360 upregulated genes and 2503 downregulated genes. The differential gene expression patterns were clearly demonstrated through the volcano plot in **Figure 3B**, while the heatmap in **Figure 3C** illustrates the expression levels of representative MetS DEGs in each sample. GSEA enrichment analysis showed downregulation in the KEGG MEDICUS REFERENCE BMP SIGNALING PATHWAY, KEGG MEDICUS REFERENCE CCR2 GNB G PI3K NFKB SIGNALING, and KEGG MEDICUS REFERENCE CCR CXCR GNB G PI3K RAC SIGNALING pathways (**Figure 3D**). WGCNA analysis, by calculating network scale independence and average connectivity, determined the optimal soft threshold setting. The data in **Figures 3E, F** indicate that the most stable network topology was achieved with a soft threshold of 11.

In **Figures 3G, H**, hierarchical clustering analysis was used to show the correlation between samples and modules, revealing significant differences in gene expression patterns between the Mets and normal groups (HC). Based on clustering, the samples were clearly divided into two groups. **Figure 3I** presents the classification results of module characteristic genes, where the module dendrogram was clustered according to the expression features of the modules. Correlation analysis further revealed the relationship between modules and clinical features, with the pink module showing the strongest negative correlation with MetS (r = -0.34, P = 0.01) as shown in **Figure 3J, K**.

### 3.2 Key gene screening and enrichment analysis of MIRI combined with MetS

**Figure 4:**
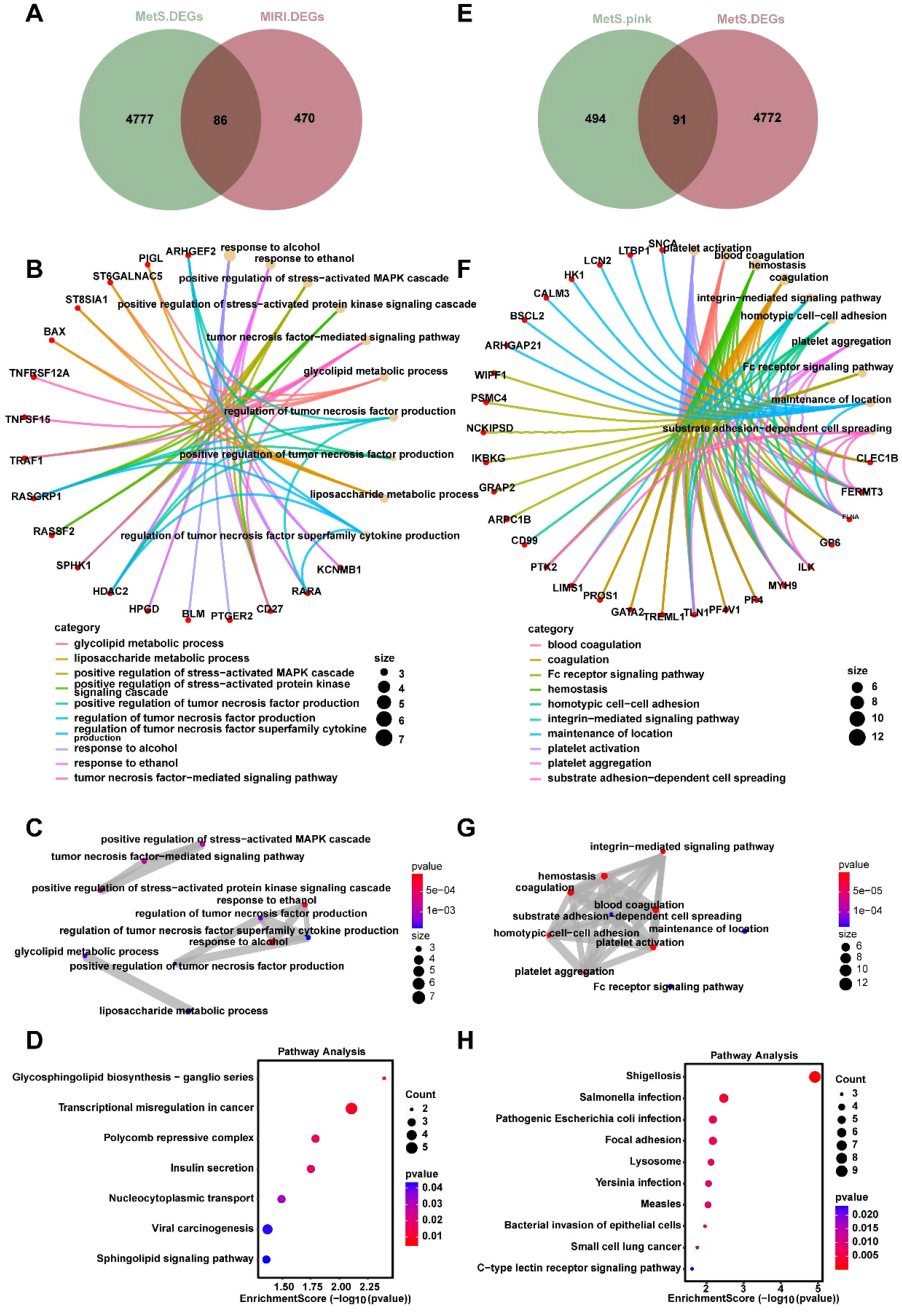
Joint enrichment analysis of biological processes and signaling pathways for MetS and MIRI. **(A)** Venn diagram of the overlapping DEGs between the MetS and MIRI groups. **(B)** GO enrichment analysis of biological processes for the intersecting genes between MetS and MIRI. **(C)** GO pathway enrichment analysis of significantly enriched pathways involving these intersecting genes. **(D)** KEGG enrichment analysis of significantly enriched pathways for the overlapping genes. **(E)** The DEGs of the “pink module” in the MetS group. **(F)** GO analysis revealing the biological processes enriched by the genes in the pink module. **(G)** GO enrichment analysis of pathway enrichment for the genes in the pink module. **(H)** KEGG enrichment analysis of the significantly enriched pathways for these genes.

To investigate the mechanisms through which MetS influences MIRI, we present a total of 4777 DEGs in the MetS group, while the MIRI group contains 470 DEGs. Among the DEGs in both groups, 86 genes are differentially expressed in both (**Figure 4A**), indicating a partial overlap of genes between the MetS and MIRI groups. Based on the intersecting genes shown in **Figure 4A**, we performed Gene Ontology (GO) analysis. **Figures 4B, C** highlight several significantly enriched biological processes, among which “positive regulation of stress-related MAPK cascade” and “glycolytic metabolic process” are most prominent. These processes play crucial roles in cellular stress responses and energy metabolism. These signals suggest that immune regulation and cellular stress response are key biological mechanisms within the intersecting genes of MIRI and MetS. **Figure 4D** presents the results of KEGG enrichment analysis, identifying significant pathways such as “lipid and carbohydrate metabolism” and “sphingolipid biosynthesis,” which may play essential roles in the pathogenesis of both MIRI and MetS. Further analysis of the MetS group was conducted, focusing on the DEGs in the “pink module” (**Figure 4E**), followed by GO and KEGG enrichment analysis. GO analysis of the pink module genes revealed that the enriched biological processes include “leukocyte adhesion” and “extracellular matrix interaction with immune cells,” both of which are closely related to immune responses and intercellular interactions (**Figure 4F**). The enrichment levels and functional characteristics of these genes further support their potential importance in disease progression. A more refined analysis of the pathways associated with the pink module revealed significant enrichment in the “integrin-mediated signaling pathway” and “platelet activation signaling pathway,” suggesting that these modules may involve crucial biological processes such as blood coagulation and cell adhesion (**Figure 4G**). Additionally, **Figure 4H** illustrates the KEGG pathway enrichment results within the pink module, highlighting pathways related to bacterial infections and immune responses, such as “Salmonella infection” and “pathogenic bacterial infections.” The enrichment of these pathways suggests that genes associated with immune responses and infections may play a pivotal role in the shared mechanisms of MIRI and MetS.

### 3.3 Construction of the MIRI diagnostic model and validation of its performance

**Figure 5:**
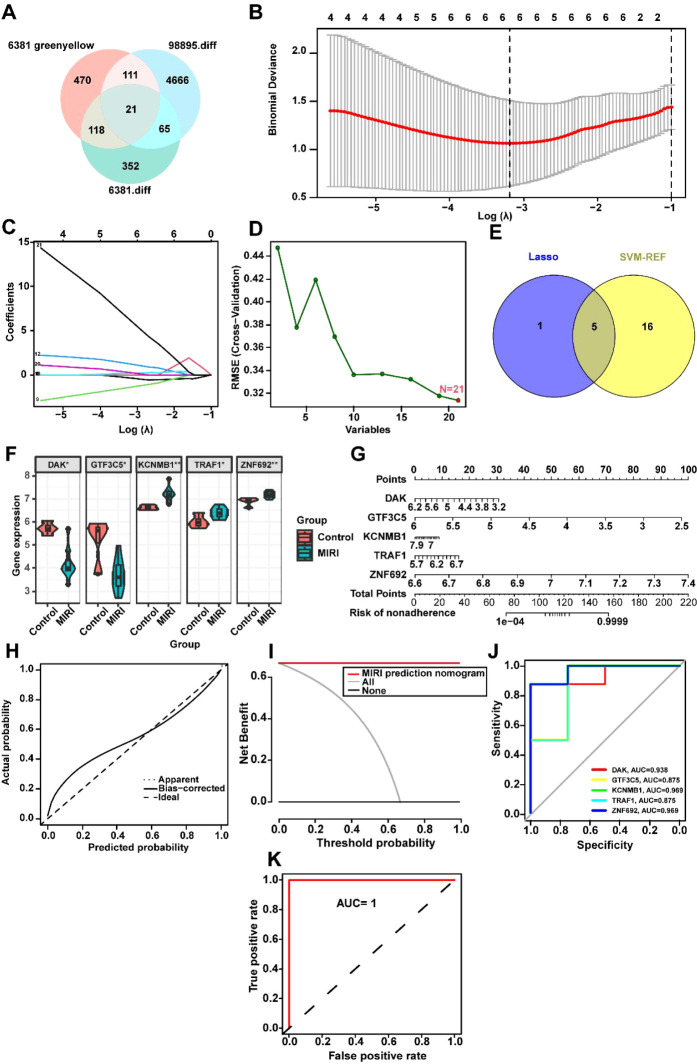
Machine Learning-Driven Identification and Validation of MIRI Diagnostic Biomarkers. **(A)** Intersectional analysis of hypoxia-responsive genes: Venn diagram identifies 21 overlapping DEGs between GSE6381 (human cardiomyocytes, n=15) and GSE98895 (murine left ventricle, n=8) datasets. **(B)** Regularization parameter optimization: Ten-fold cross-validation curve showing mean squared error (y-axis) versus log(λ) (x-axis). Optimal λ (0.032, dotted line) selected where deviance reaches minimum (error bars = ± 1 SD across 100 bootstrap iterations). **(C)** LASSO coefficient trajectories: Shrinkage of 21 candidate genes (colored lines) with increasing penalty. **(D)** SVM-RFE feature ranking: Recursive elimination plot showing 21 genes sorted by elimination order (right to left). No features excluded (100% retained) based on 5% accuracy loss threshold (dashed horizontal line). **(E)** Consensus biomarker selection: Intersection of LASSO (n=5) and SVM-RFE (n=21) outputs identifies five core genes (DAK, GTF3C5, KCNMB1, TRAF1, ZNF692). **(F)** Diagnostic biomarker expression: Violin plots of five-gene signature in GSE6381. Heatmap inset shows z-score normalized expression across 23 MIRI vs. 12 control samples. **(G)** Clinical translation nomogram: Points-to-risk conversion model integrating biomarker expression with baseline covariates (age >50, diabetes status). Calibration slope = 0.94 (95%CI 0.89-0.99) via bootstrap. **(H)** Model calibration: Observed vs. predicted MIRI probability (Loess-smoothed, bandwidth=0.75) with Brier score = 0.11 (null model = 0.25). Diagonal reference line indicates perfect calibration. **(I)** Decision curve analysis: Net benefit (y-axis) across probability thresholds (x-axis, 0-1). Combined model (red) outperforms treat-all (gray) and treat-none (black) strategies between 12-89% threshold probabilities. **(J)** Individual biomarker performance: Time-dependent ROC curves at 24h post-reperfusion.AUC values: KCNMB1 (0.969), ZNF692 (0.969), DAK (0.938), GTF3C5 (0.875), TRAF1 (0.875). Shaded regions = 95% CIs. **(K)** Combinatorial diagnostic power: Composite ROC of five-gene panel achieves perfect discrimination (AUC=1.00, DeLong test p=2.3×10^7^ vs. best single marker). Internal validation via 80:20 split (n=100 iterations). *p<0.05; **p<0.01.

To identify key biomarkers associated with MIRI, we performed a Venn diagram analysis of critical genes in the GSE6381 and GSE98895 datasets, resulting in the identification of 21 common variables (**Figure 5A**, **Supplementary Table 1**). Subsequently, the optimal regularization parameter was determined through cross-validation, which ensured the stability of the selection process (**Figure 5B**). The Lasso regression model coefficient plot was provided, illustrating the relationship between the six selected key genes and their respective coefficients (**Figure 5C**). Additionally, using SVM-REF to screen these 21 variables, this method did not exclude any variables (**Figure 5D**). The intersection of variables obtained from both LASSO and SVM-REF analyses identified five genes: DAK, GTF3C5, KCNMB1, TRAF1, and ZNF692 (**Figure 5E**). The expression levels of these five variables in the GSE6381 dataset were presented, with DAK and GTF3C5 showing low expression in MIRI, while KCNMB1, TRAF1, and ZNF692 exhibited high expression (**Figure 5F**). A nomogram was constructed using these five variables to calculate the non-compliance risk based on the identified biomarkers, displaying the corresponding scores and overall risk (**Figure 5G**). **Figures 5H–K** focus on model performance, where **Figure 5H** shows the calibration curve comparing ideal and actual probabilities with minimal error, and **Figure 5I** demonstrates the higher net benefit of the MIRI model at various thresholds. **Figure 5J** compares the ROC area under the curve (AUC) values of five biomarkers: DAK (0.938), GTF3C5 (0.875), KCNMB1 (0.969), TRAF1 (0.875), and ZNF692 (0.969). Finally, **Figure 5K** indicates that the combined analysis of DAK, GTF3C5, KCNMB1, TRAF1, and ZNF692 yields an AUC of 1 for the ROC curve, suggesting that these genes collectively exhibit excellent diagnostic efficacy for MIRI (AUC > 0.8).

**Figure 6:**
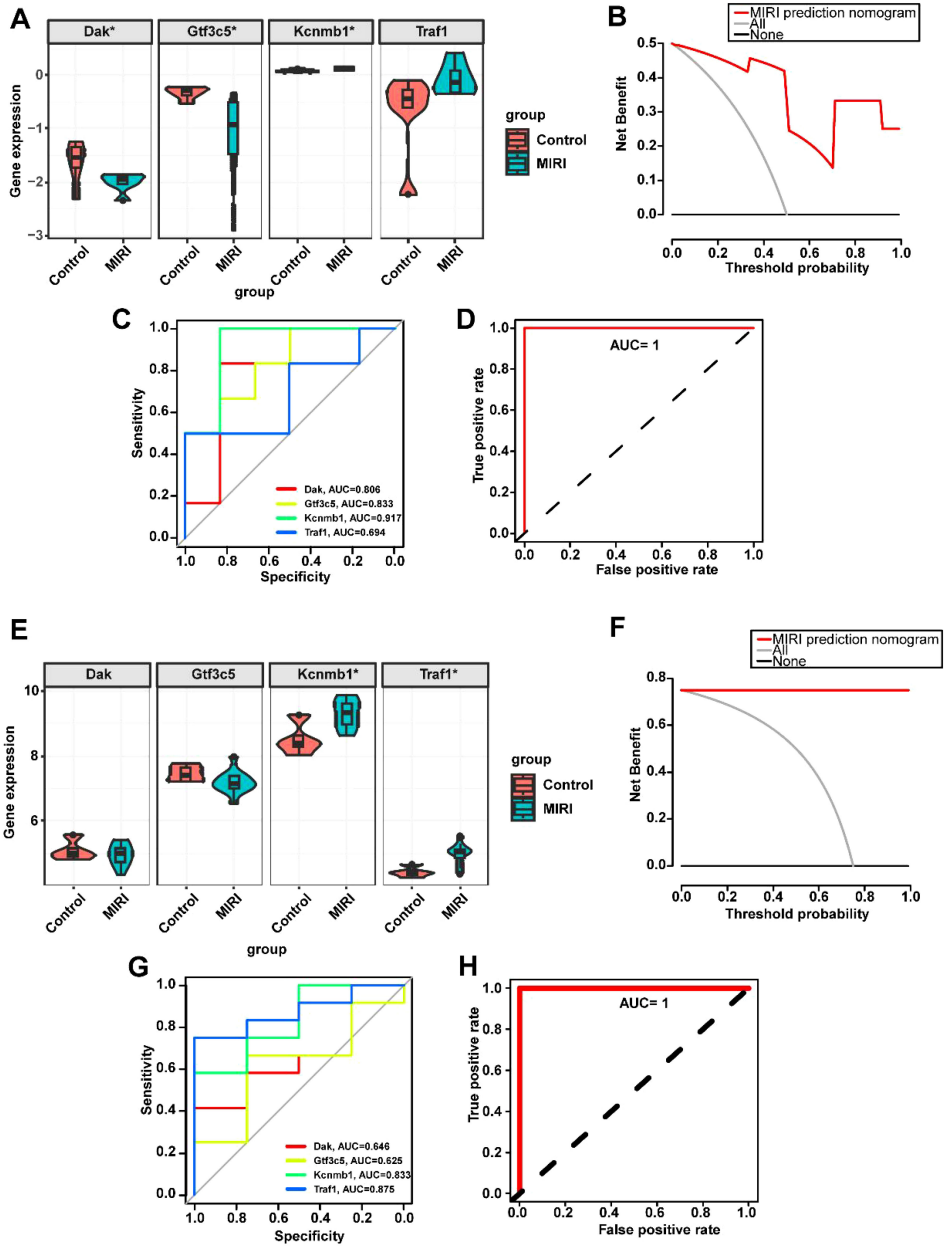
Validation of key gene expression in the MIRI mouse datasets GSE108940 and GSE160516. **(A)** Expression levels of key genes in GSE108940. **(B)** Decision curve analysis for predicting MIRI using key genes in GSE108940. **(C)** ROC curves of individual key genes in GSE108940. **(D)** Combined ROC curve of key genes in GSE108940. **(E)** Expression levels of key genes in GSE160516. **(F)** Decision curve analysis for predicting MIRI using key genes in GSE160516. **(G)** ROC curves of individual key genes in GSE160516. **(H)** Combined ROC curve of key genes in GSE160516. *p < 0.05.

To validate the aforementioned results, we downloaded the mouse datasets GSE108940 and GSE160516 related to MIRI and performed verification of the key genes. In both the GSE108940 and GSE160516 datasets, we identified four mouse homologous genes—Dak, Gtf3c5, Kcnmb1, and Traf1. Consistent with our previous findings, Dak was found to be downregulated in both GSE108940 and GSE160516, while Gtf3c5, Kcnmb1, and Traf1 were upregulated in both datasets (**Figures 6A, E**). The clinical decision curve (DCA) analysis of the MIRI model constructed by Dak, Gtf3c5, Kcnmb1, and Traf1 shows a larger area under the curve, indicating a superior clinical decision-making capacity (**Figure 6B, F**). Additionally, the ROC curve analysis revealed that the AUC for individual genes ranged from a minimum of 0.694 to a maximum of 0.917 (**Figures 6C, G**). The combined ROC curve of Dak, Gtf3c5, Kcnmb1, and Traf1 reached an AUC of 1, suggesting that the combined analysis of these four genes offers superior diagnostic performance for MIRI (**Figures 6D, H**).

### 3.4 Analysis of the impact of key genes on immune infiltration in MIRI and MetS

**Figure 7:**
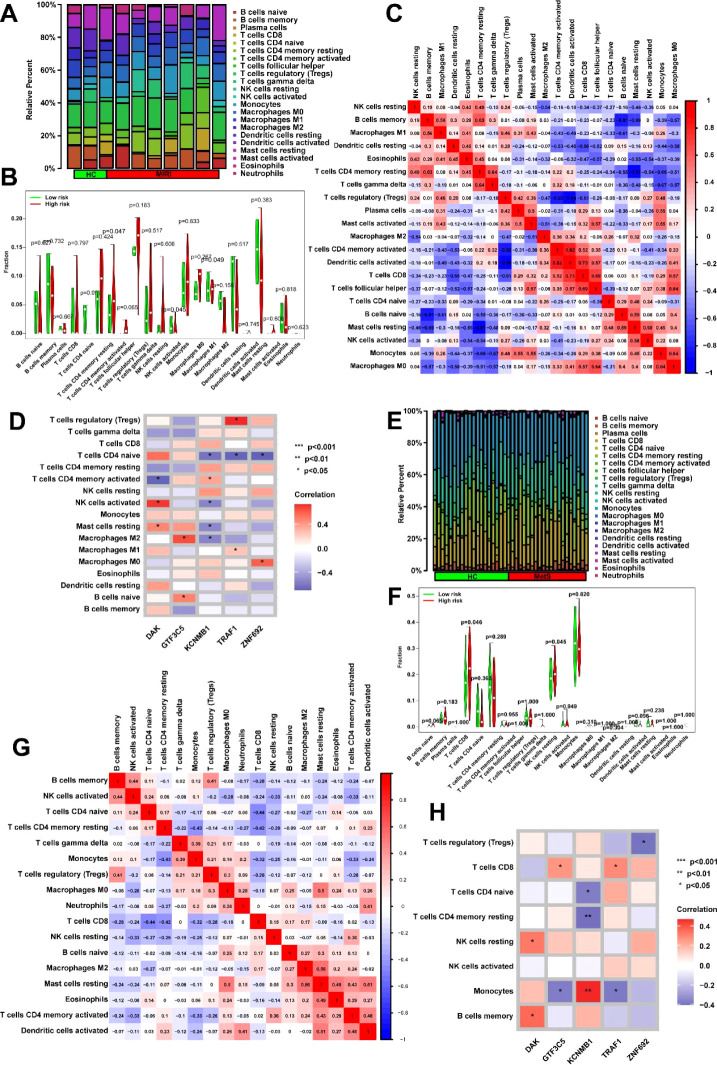
Immune microenvironment dynamics and key gene interactions in MIRI and metabolic syndrome. **(A)** Immune landscape remodeling in MIRI. Left: CIBERSORTx analysis reveals altered immune cell proportions in MIRI, with increased infiltration of CD4+ memory T cells, activated NK cells, and pro-inflammatory M1 macrophages compared to controls. Right: t-SNE visualization highlights distinct immune clustering in MIRI samples (arrows indicate enrichment of pro-inflammatory populations). **(B)** Quantitative immune signature in MIRI. Box plots demonstrate elevated infiltration levels of three key immune populations (CD4+ memory T cells, activated NK cells, M1 macrophages) in MIRI, with error bars reflecting intergroup distribution trends. **(C)** Immune cell coordination network in MIRI. Correlation heatmap reveals dynamic immune interactions: a strong positive axis between activated NK cells and resting CD4+ memory T cells (red module), contrasted with negative correlations between activated CD4+ T cells and M2 macrophages (blue module), suggesting disrupted immune activation-suppression balance. **(D)** Gene-immune regulatory axes in MIRI. Network diagram illustrates positive associations between KCNMB1 and resting NK cells (orange edges), and between TRAF1 and activated CD4+ T cell infiltration (purple edges). Node size reflects genes’ hub roles in the regulatory network. **(E)** MetS-specific immune phenotype. Stacked bar plots show increased cytotoxic immune populations (CD8+ T cells, NK cells, M1 macrophages) and dendritic cell activation in MetS compared to controls. **(F)** Stratified immune activation in MetS. Violin plots compare resting CD8+ T cell and NK cell infiltration between groups, demonstrating broader activation heterogeneity in MetS (dashed lines mark median shifts). **(G)** MetS immune crosstalk network. Circos plot identifies synergistic interactions between memory B cells and activated NK cells (red ribbons), alongside antagonistic relationships between activated CD4+ T cells and neutrophils (blue ribbons). Gray connections denote non-significant associations. **(H)** Key Gene-Driven Immune Regulation in MetS. Circular heatmap highlights TRAF1’s dual associations with activated NK cells and CD4+ T cells (outer gene ring vs. inner immune cell sectors), suggesting its role in coordinating immune responses. *p<0.05; **p<0.01; ***p<0.001.

Given that the DEGs enrichment analysis of MIRI and Mets in **Figure 4** reveals significant associations with immune response, we then analyzed the immune cell infiltration of key genes in MIRI and MetS. Significant differences in immune cell infiltration were observed between the normal and MIRI groups, with immune cell infiltration being more active in the MIRI group compared to the control group. Notably, higher infiltration levels of CD4 memory T cells, activated NK cells, and M1 macrophages were observed in the MIRI group. These findings suggest that immune responses may play an important role in the progression of MIRI (**Figures 7A, B**). In MIRI, immune cells showed interactions, with a strong positive correlation observed between activated NK cells and resting CD4 memory T cells, as well as between activated dendritic cells and activated CD4 memory T cells, indicating that these cells may cooperate in the immune response. Conversely, negative correlations were found between activated CD4 memory T cells and certain immune cell populations (such as M2 macrophages and activated mast cells), suggesting the potential presence of immune suppression or regulatory mechanisms (**Figure 7C**). Key genes were involved in the regulation of immune cell levels, with KCNH5 showing a positive correlation with resting NK cells, while TRAF1 was associated with activated CD4 memory T cells. This influence may contribute to the differential levels of immune cells in MIRI (**Figure 7D**).

In MetS, compared to the control group, the MetS group exhibited higher levels of immune cell infiltration, particularly in T cells CD8, NK cells, M1 macrophages, and dendritic cells (**Figure 7E**). Statistical analysis of immune cell infiltration further confirmed the significantly increased infiltration levels of resting CD8 T cells and NK cells in the MetS group (**Figure 7F**). Correlation analysis between immune cells revealed a strong positive correlation between memory B cells and activated NK cells, suggesting a potential cooperative role of these cells in immune responses. On the other hand, activated CD4 memory T cells showed a negative correlation with other immune cells (such as M2 macrophages and neutrophils) (**Figure 7G**). Correlation analysis of key genes with immune cells showed significant associations between multiple key genes and the infiltration of immune cell populations. For instance, the gene TRAF1 was positively correlated with activated NK cells and also exhibited a strong correlation with activated CD4 memory T cells (**Figure 7H**). Through these gene-immune cell interactions, the levels of immune cell infiltration in MetS are regulated.

### 3.5 Biological pathways regulated by key genes and drug prediction

**Figure 8:**
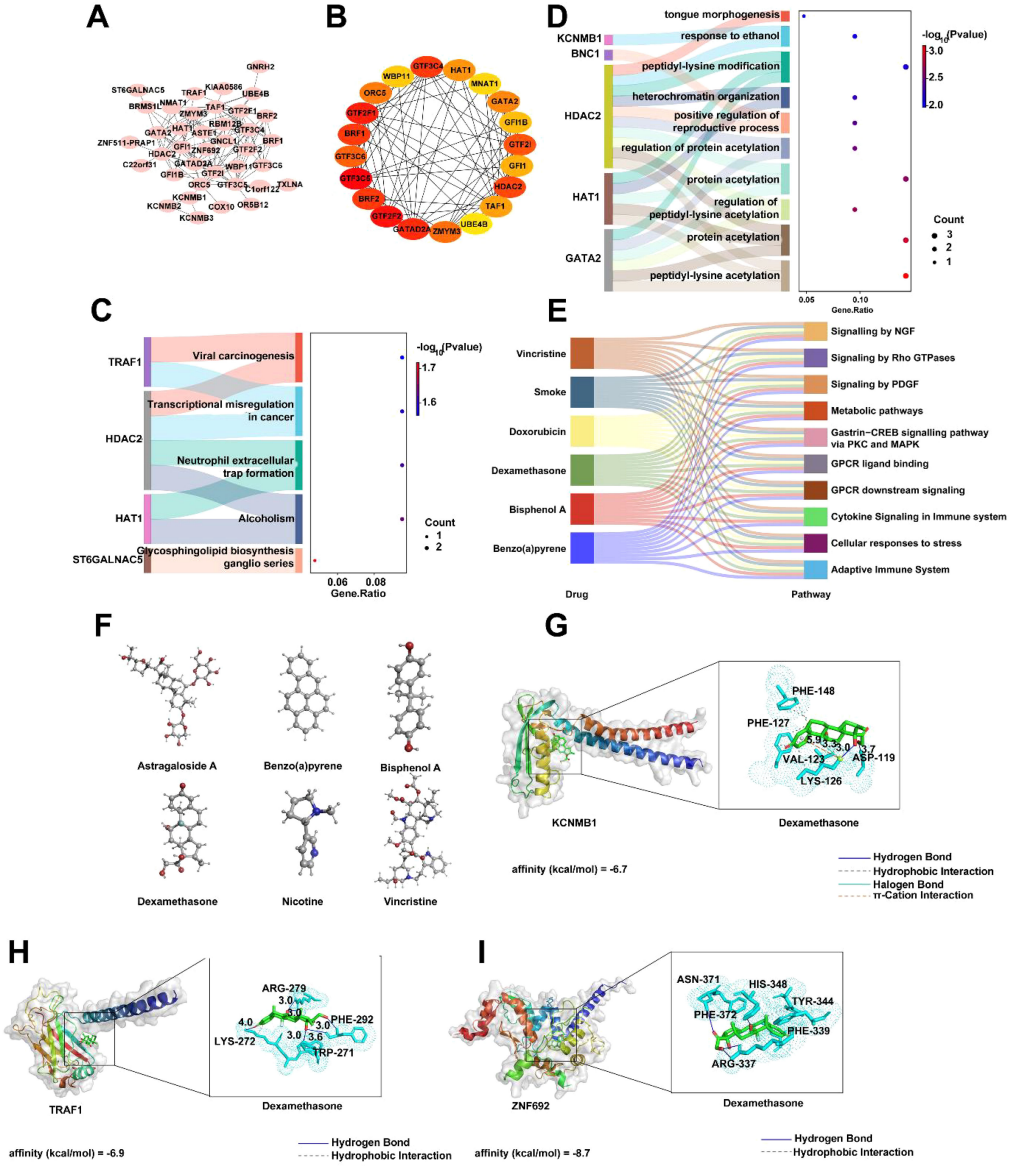
PPI Network and Associated Analysis of Key Genes DAK, GTF3C5, KCNMB1, TRAF1, and ZNF692. **(A)** PPI network of DAK, GTF3C5, KCNMB1, TRAF1, and ZNF692 constructed using the STRING database. **(B)** Network interaction results of the top 20 most important genes identified based on the PPI network, using the CytoHubba plugin. **(C)** GO enrichment analysis of the top 20 important genes. **(D)** KEGG enrichment analysis results of the top 20 important genes. **(E)** Six drugs associated with the key genes identified based on CTD drug prediction results. **(F)** Molecular structures of the six drugs. **(G-I)** Representative docking pair combinations with strong binding energy from the drug.

To further analyze the roles of key genes DAK, GTF3C5, KCNMB1, TRAF1, and ZNF692, we constructed a PPI network for these genes using the STRING database, which displayed the interactions among the genes. The network consists of 38 nodes and 114 edges (**Figure 8A**). Based on the PPI network, the top 20 most significant genes were identified using the Cytohubba plugin, including GTF3C5, GTF2F2, GTF2F1, GATAD2A, and GTF3C4 (**Figure 8B**). We then analyzed the biological functions impacted by these 20 important genes, primarily focusing on pathways such as viral carcinogenesis, transcriptional regulation in cancer, and neutrophil exosome formation, which are closely related to immune response molecular mechanisms (**Figure 8C**). KEGG enrichment analysis revealed several pathways, including peptide-lysine modification, heterochromatin organization, and protein acetylation, indicating the involvement of these genes in intracellular signaling and gene expression regulation (**Figure 8D**). Additionally, we explored drug predictions for the key genes in the CTD. Based on the overlap of gene interactions, six related drugs were identified, such as vincristine, tobacco, doxorubicin, and bisphenol A, which influence pathways such as Signaling by NGF, Signaling by Rho GTPases, and Signaling by PDGF, suggesting potential therapeutic and toxic effects (**Figure 8E**). The molecular structures of these six drugs were also presented to assist in understanding their chemical compositions (**Figure 8F**). Subsequently, docking analysis revealed that KCNMB1, TRAF1, and ZNF692 all exhibited strong binding affinity with dexamethasone. The affinity score of dexamethasone with KCNMB1 was -6.7 kcal/mol, where hydrogen bonds (blue solid lines) were formed between dexamethasone and ASP-119, LYS-126, Pi-cation interactions (orange dashed lines) and halogen bonds (cyan solid lines) with LYS-126, as well as hydrophobic interactions (gray dashed lines) with VAL-123, LYS-126, PHE-127, and PHE-148 (**Figure 8G**). The affinity score of dexamethasone with TRAF1 was -6.9 kcal/mol, with hydrogen bonds formed between dexamethasone and LYS-272, ARG-279, PHE-292, and TRP-271 (blue solid lines), and hydrophobic interactions with LYS-272 (gray dashed lines) (**Figure 8H**). The affinity score of dexamethasone with ZNF692 was -8.7 kcal/mol, with hydrogen bonds formed between dexamethasone and ASN-371, HIS-348, ARG-337 (blue solid lines), and hydrophobic interactions with PHE-339, TRY-344, and HIS-348 (gray dashed lines) (**Figure 8I**).

### 3.6 Dexamethasone effect on MIRI in an *in vitro* cell model

**Figure 9:**
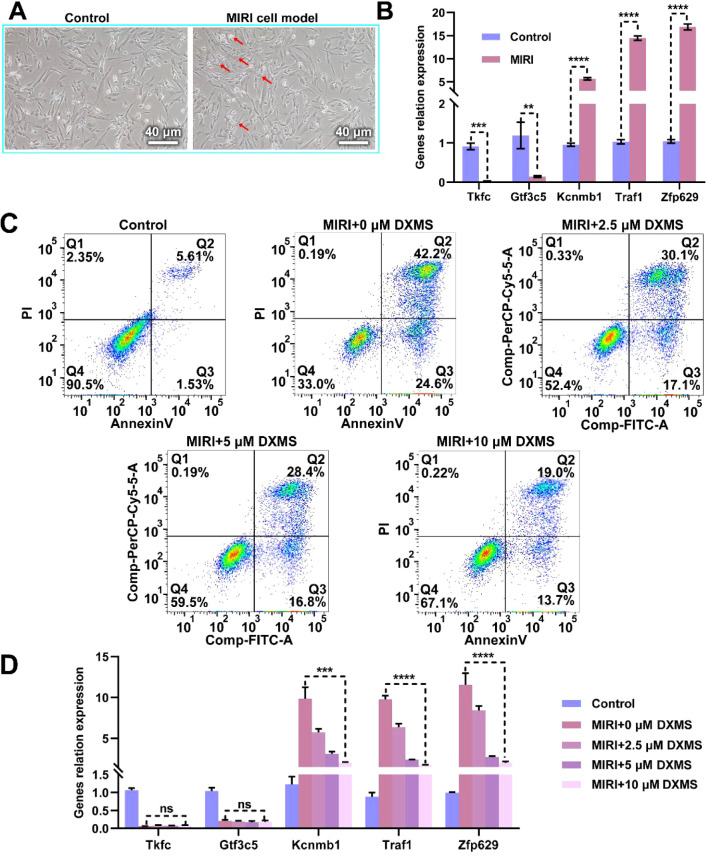
Effect of Dexamethasone on Hypoxia-Induced H9C2 Cell MIRI Model. **(A)** Microscopic images comparing the H9C2 cell MIRI model induced by hypoxic conditions with the normal control group. The left panel shows the normal control group, and the right panel shows the MIRI model group, scale bar = 20 μm. **(B)** Expression levels of key genes (Dak, Gtf3c5, Kcnmb1, Traf1, Znf692) in the MIRI model. **(C)** Effect of dexamethasone on cell apoptosis in the MIRI model. The apoptosis rates of different treatment groups (control, MIRI+0μM dexamethasone, MIRI+2.5μM dexamethasone, MIRI+5 μM dexamethasone, MIRI+10 μM dexamethasone) were analyzed by flow cytometry. **(D)** The changes in RNA levels of key genes (Dak, Gtf3c5, Kcnmb1, Traf1, Znf692) in the MIRI model under different concentrations of dexamethasone treatment. ns: not significant; **p < 0.01; ***p < 0.001; ****p < 0.0001.

In this study, we first induced a MIRI model in H9C2 cells through hypoxic treatment and observed typical changes in cell morphology (**Figure 9A**). We then analyzed the expression of key genes Dak, Gtf3c5, Kcnmb1, Traf1, and Znf692 in this model (**Figure 9B**). The results showed that the expression levels of Dak and Gtf3c5 were significantly lower in the MIRI group compared to the normal control group, while Kcnmb1, Traf1, and Znf692 were significantly higher. Subsequently, we added different concentrations of dexamethasone (0 μM, 2.5 μM, 5 μM, and 10 μM) and observed cell apoptosis (**Figure 9C**). Compared to the MIRI group, as the dexamethasone concentration increased, the number of apoptotic cells in the treatment groups gradually decreased, but it remained higher than in the normal control group, suggesting that dexamethasone could alleviate cell apoptosis, with higher concentrations showing more pronounced effects, though it did not completely reverse the apoptosis caused by MIRI. Finally, we measured the expression of key genes in the different drug treatment groups. The results indicated that the expression levels of Dak and Gtf3c5 remained unchanged after dexamethasone treatment, while the levels of Kcnmb1, Traf1 and Znf692 gradually decreased with increasing dexamethasone concentration (**Figure 9D**). These findings suggest that dexamethasone treatment significantly regulated the expression of these genes, further supporting its protective effect on the MIRI cell model. The results indicate that dexamethasone can suppress MIRI-induced cell apoptosis to some extent and exert its effect by regulating the expression of related genes.

## 4 Discussion

MIRI is a myocardial injury induced by the restoration of blood flow, and its underlying mechanisms are still not fully understood (36, 37). Recent studies suggest that MetS, as an important risk factor for cardiovascular diseases, may exacerbate MIRI in multiple ways (10, 38). For instance, MetS amplifies oxidative stress and mitochondrial dysfunction during reperfusion, which aligns with our hypothesis of shared pathways between MetS and MIRI (39). However, the specific relationship between MetS and myocardial ischemia-reperfusion, along with the molecular mechanisms involved, remains unclear (40, 41). To explore this potential association, this study utilized genomic analysis and identified significant overlaps between MIRI and MetS. Specifically, despite displaying different gene expression patterns, we found 86 overlapping genes that were significantly differentially expressed in both MIRI and MetS through DEG analysis. These findings directly address a critical knowledge gap by identifying TRAF1 and KCNMB1 as potential dual-purpose therapeutic targets, offering a molecular basis for developing therapies that simultaneously mitigate metabolic dysfunction and reperfusion injury. This discovery provides a new perspective on understanding the molecular mechanisms linking MetS and MIRI and may offer a theoretical foundation for future therapeutic strategies.

Given the role of MetS in myocardial ischemia-reperfusion, we screened the most critical genes influencing their interaction for predicting the onset of myocardial ischemia-reperfusion. This study constructed a diagnostic model for MIRI based on key genes such as DAK, GTF3C5, KCNMB1, TRAF1, and ZNF692, and validated the model’s effectiveness using independent datasets from mice and rats. Through DCA and ROC curves, we found that the diagnostic model exhibited high accuracy in MIRI diagnosis, and the combined analysis of these five genes resulted in an ROC curve area of 1, indicating excellent clinical predictive ability. This multi-gene approach holds immediate translational value: It provides a framework for developing non-invasive blood tests (e.g., circulating RNA panels) to identify MetS patients at high risk of MIRI prior to revascularization procedures, enabling personalized prophylactic interventions. Our multi-gene approach demonstrates superior predictive performance compared to previous studies focusing on non-joint diagnostic biomarker development (35). This result not only provides important evidence for the early diagnosis of MIRI but also serves as a valuable reference for related clinical research.

Currently, drug therapy is one of the primary methods to reduce MIRI (42, 43). Common drugs include antioxidants, anti-inflammatory agents, and cytoprotective drugs. Dexamethasone, a widely used glucocorticoid, has been shown to have anti-inflammatory and immunomodulatory effects (44, 45). A recent clinical trial by Jimenez-Valero reported that dexamethasone reduced post-reperfusion infarct size by 3.3% in STEMI patients, consistent with our observed anti-apoptotic effects (46). Our findings extend these clinical observations by proposing a gene-guided application: Dexamethasone may be particularly beneficial in MetS patients with upregulated Traf1/Kcnmb1 expression, suggesting a precision medicine approach for patient stratification. This study explored the therapeutic effect of dexamethasone in the MIRI model. After treating with different concentrations of dexamethasone, we observed that dexamethasone significantly reduced cell apoptosis induced by MIRI, and the therapeutic effect progressively improved with increasing dexamethasone concentration. However, although dexamethasone treatment alleviated MIRI-induced cell apoptosis, its effect was not sufficient to fully reverse the damage, suggesting that its protective effect in treating MIRI may have certain limitations. Additionally, we further analyzed the impact of dexamethasone on the expression of key genes (such as Dak, Gtf3c5, Kcnmb1, Traf1, and Znf692) in the MIRI model. The results showed that dexamethasone might alleviate myocardial ischemia-reperfusion damage to some extent by inhibiting the expression of KCNMB1, TRAF1, and ZNF692 genes. This gene regulatory pattern is similar to the mechanism of novel MIRI therapies such as NLRP3 inflammasome inhibitors (47), suggesting potential synergistic therapeutic strategies. Therefore, while dexamethasone has therapeutic potential, its clinical application in MIRI treatment requires further validation and may necessitate more precise therapeutic approaches.

Although this study provides new molecular insights into the relationship between MetS and myocardial ischemia-reperfusion and proposes an effective diagnostic model, some limitations remain. First, the study mainly relied on animal models and public datasets for analysis, lacking validation with human samples, which may limit the generalizability of the results. To bridge this gap, future work should prioritize clinical translation through: (1) Prospective validation of the five-gene panel in human cohorts undergoing cardiac surgery, and (2) Pharmacodynamic studies assessing dexamethasone’s gene-modulatory effects in MetS patients. Second, while dexamethasone demonstrated certain efficacy in the cell model, its clinical effectiveness still needs to be validated through more clinical trials. The molecular mechanisms of MIRI are highly complex, and future research should incorporate more experimental data to further investigate other potential biomarkers and therapeutic targets, providing a more comprehensive foundation for improving the clinical treatment of MIRI.

## 5 Conclusion

The MIRI diagnostic model based on key genes such as DAK, GTF3C5, KCNMB1, TRAF1, and ZNF692 constructed in this study has high clinical predictive ability and can provide important basis for early diagnosis of MIRI. However, the translational relevance of these biomarkers requires further validation in human myocardial specimens. Future studies should verify their expression patterns in human ischemic heart tissues through autopsy samples or endomyocardial biopsies to bridge the gap between animal models and clinical applications. In addition, the application of dexamethasone in the MIRI model has demonstrated its potential to alleviate cell apoptosis, although the therapeutic effect is limited, it still provides ideas for future treatment strategies. Importantly, the species-specific differences in drug response necessitate subsequent validation in human cardiomyocytes or cardiac organoids to assess clinical feasibility. In summary, this study reveals a potential association between MetS and MIRI, providing a new molecular mechanism perspective for understanding the role of MetS in MIRI.

## 6 Perspectives

Future studies should validate the hub genes (DAK, GTF3C5, KCNMB1, TRAF1, ZNF692) in human MetS-MIRI cohorts to refine diagnostic and prognostic utility. The limited efficacy of dexamethasone underscores the need for synergistic therapies targeting both KCNMB1-related ion channels and TRAF1/ZNF692-driven inflammation. Multi-omics approaches could dissect how MetS exacerbates MIRI via metabolic-immune crosstalk, while organoid models mimicking MetS-MIRI comorbidity may accelerate drug discovery. Prioritizing human tissue validation and combinatorial strategies will bridge mechanistic insights to precision therapies, addressing unmet clinical needs in patients with dual metabolic and cardiovascular risks.

## Data Availability

The raw data supporting the conclusions of this article will be made available by the authors, without undue reservation.
